# Temperature-Ion-pH Triple Responsive Gellan Gum as In Situ Hydrogel for Long-Acting Cancer Treatment

**DOI:** 10.3390/gels8080508

**Published:** 2022-08-15

**Authors:** Shuwen Zhou, Xinmeng Zheng, Ke Yi, Xuancheng Du, Cheng Wang, Pengfei Cui, Pengju Jiang, Xinye Ni, Lin Qiu, Jianhao Wang

**Affiliations:** 1 School of Pharmacy, Changzhou University, Changzhou 213164, China; 2 The Affiliated Changzhou No.2 People’s Hospital of Nanjing Medical University, Changzhou 213003, China; 3 School of Physics, Shandong University, Jinan 250100, China

**Keywords:** hydrogel, responsive, gellan gum, doxorubicin, cancer

## Abstract

Background: Promising cancer chemotherapy requires the development of suitable drug delivery systems (DDSs). Previous research has indicated that a hydrogel is a powerful DDS for tumor therapy and holds great potential to offer a feasible method for cancer management. Methods: In this study, glutathione-gellan gum conjugate (GSH-GG) was synthesized through chemical reaction. Doxorubicin hydrochloride (DOX) was loaded into GSH-GG to accomplish DOX-loaded GSH-GG. The properties, injectability, drug release, and in vitro and in vivo anticancer effects of DOX-loaded GSH-GG were tested. Results: DOX-loaded GSH-GG showed a temperature-ion dual responsive gelling property with good viscosity, strength, and injectability at an optimized gel concentration of 1.5%. In addition, lower drug release was found under acidic conditions, offering beneficial long-acting drug release in the tumor microenvironment. DOX-loaded GSH-GG presented selective action by exerting substantially higher cytotoxicity on cancer cells (4T1) than on normal epithelial cells (L929), signifying the potential of complete inhibition of tumor progression, without affecting the health quality of the subjects. Conclusions: GSH-GG can be applied as a responsive gelling material for delivering DOX for promising cancer therapy.

## 1. Introduction

Cancer is a lethal disease, causing substantial mortality worldwide [1,2]. Over 1.9 million new cases and 0.6 million cancer-related deaths were estimated in the United States alone for 2022 [3]. This severe health burden desperately calls for the development of effective anticancer approaches. Chemotherapy remains one of the most widely adopted treatment options in current cancer management [4,5]. However, the application of chemotherapeutic drugs still faces significant challenges, including low bioavailability [6], intense pain, and severe side effects [7]. To overcome these problems, pharmaceutical researchers actively seek to formulate efficient strategies.

So far, it has been generally recognized that drug delivery systems (DDSs) that can realize controlled drug release are suitable for efficacious cancer therapy [8,9]. Therefore, various types of DDSs, such as micelles [10], liposomes [11], and hydrogels [12,13], have been successfully devised using synthetic and/or natural materials. Among them, hydrogels are emerging as promising DDS for cancer chemotherapy, owing to their structures that are capable of retaining a high water content and maintaining a three-dimensional network, which allows encapsulation of different drugs. For example, Amano et al. developed a nano-sized hyaluronic acid (HA)-based cisplatin-loaded hydrogel, which showed a significant therapeutic effect in both the xenograft and allograft models [14]. Wang et al. proposed a locally injectable endostatin-loaded hydrogel to remodel the tumor microenvironment through anti-angiogenic effects [15]. These studies underline the potential and feasibility of hydrogels in cancer treatment.

Since hydrogels show high gelling temperature, their local injections require additional heating prior to administration [16,17]. Moreover, they also need the aid of other cross-linking agents to initiate the gelation process [18,19]. These characteristics make applying these hydrogels complicated and unfavorable for in vivo anticancer activity. Gellan gum (GG) is a naturally originated anionic exopolysaccharide that is widely used as a drug carrier, due to its non-toxicity and biodegradability [20,21]. In particular, GG showed an ion-responsive gelation property upon contacting common metal ions (such as K^+^, Ca^2+^, and Mg^2+^) in physiological environments [22]. However, the hydrogel formed by GG often exhibits weak mechanical strength and high gelation temperature, severely hindering its further application [23,24]. Therefore, proper modification of GG could reduce its gelling temperature to be appropriate for the human body in vivo, while maintaining its ion-responsive nature. Such an alteration is essential to improve the application of GG in cancer therapy.

In our study, we firstly presented a new method of modifying GG with glutathione (GSH) to accomplish glutathione conjugated-GG (GSH-GG), which could form in situ hydrogels. Doxorubicin hydrochloride (DOX)-loaded GSH-GG showed a substantial decrease in gelation temperature, intact ion-responsive nature, and slow drug release in an acidic environment. We demonstrated the applicability of GSH-GG as a DOX-loading and in situ gelling material in vitro for selective 4T1 cancer cytotoxicity. Moreover, the in vivo r effect of the DOX-loaded GSH-GG gel on 4T1 tumor inhibition was also tested.

## 2. Results and Discussion

### 2.1. Characterization of GSH-GG

A GSH-GG conjugate was synthesized through the condensation reaction between the carboxyl group of GG and the amino group of GSH. The average yield of GG-mercaptoacetic acid coupling was 71%. The results showed that the unreacted GSH in the product could be removed by dialysis under the conditions of low temperature (4 °C), no oxygen (bubbling by N_2_), and an acidic medium (pH = 3). Following freeze-drying, a water-insoluble white product was obtained, which dissolved at a temperature above 80 °C and pH > 6, and formed a transparent hydrogel after cooling. The feasibility of this modification method and the correctness of the final product have been proven by ^1^HNMR, FT-IR, SEM, and rheological studies in our previous study [24]. The DOX can be readily loaded into the GSH-GG to obtain DOX-loaded GSH-GG by simply using the DOX solution as the solvent. 

**Figure 1:**
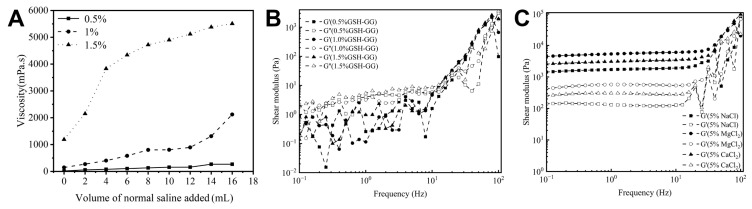
(**A**) Viscosity changes in DOX-containing GSH-GG with different concentrations after adding normal saline. (**B**) Rheological curves of the GSH-GG hydrogels prepared at different concentrations. (**C**) Rheological curves of the GSH-GG hydrogels with the concentration of 1.5% (*w*/*v*) after adding liquid of different ionic strength.

The physical and chemical properties play an important role in the properties of in situ gels [25]. First, we studied the viscosity changes in some series of concentrations of DOX-loaded GSH-GG in a normal saline environment (Figure 1A). Without normal saline, the viscosities of three kinds of DOX-loaded GSH-GG were 17, 148, and 891 mPa.s, indicating an increase in viscosity with increasing GSH-GG concentration. With the increase in the volume of normal saline, the gel viscosity of each concentration also showed a gradual upward trend, and the higher the concentration of GSH-GG, the faster the viscosity increased. After adding 16 mL of saline, the viscosity of 1.5% DOX-loaded GSH-GG reached 5510 mPa.s, while 0.5% DOX-loaded GSH-GG only had 268 mPa.s [26]. The gel strength plays a certain role in whether the in situ gel can maintain the solid state under the skin [27]. The rheology of GSH-GG hydrogels prepared at different concentrations is shown in Figure 1B. The elastic modulus (G′) and viscous modulus (G″) of GSH-GG hydrogels with concentrations of 0.5%, 1%, and 1.5% at different frequencies were measured. When the G′ of GSH-GG was greater than G″, it indicated that GSH-GG was a stable cross-linked structure and could show elastic deformation under shear. The increase in frequency decreased the G′ to less than G″. With the increase in GSH-GG concentration, the intersection point of G′ and G″ moved to the high-frequency region. It indicated a gradual increase in the hydrogels’ cross-linking degree and upshift of both G′ and G″ with increasing GSH-GG concentration. As shown in Figure 1C, the rheology of the GSH-GG hydrogels with a concentration of 1.5% (*w*/*v*) was tested in solutions of varying ionic strengths. The results showed that after adding 5% NaCl solution, G″ was greater than G′ when the frequency increased to above 80 Hz. After adding 5% MgCl_2_ or CaCl_2_ solution, G′ was always greater than G″, indicating that GSH-GG hydrogels with added MgCl_2_ or CaCl_2_ were stronger than GSH-GG hydrogels with added NaCl.

### 2.2. Effect of Temperature and Cations on Gelling Behavior of GSH-GG

**Figure 2:**
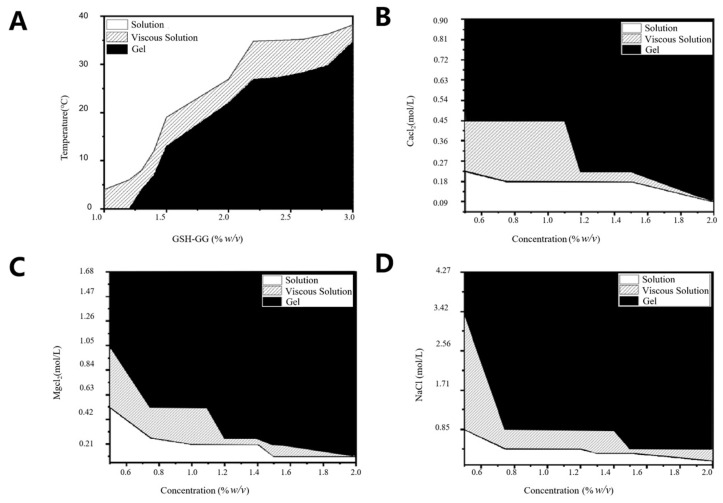
Effect of temperature (**A**) and cations ((**B**) for Ca^2+^, (**C**) for Mg^2+,^ and (**D**) for Na^+^) on gelling behavior of GSH-GG.

Figure 2 shows the phase diagram of the effect of temperature, Ca^2+^, Mg^2+^, and Na^+^ ions on the gelling behavior of DOX-loaded GSH-GG. The aqueous solution of DOX-loaded GSH-GG appeared to be clear at its low concentration (1%). However, it became viscous with declining temperature. In addition, it was also observed that when the temperature of DOX-loaded GSH-GG was higher than 11.0 °C, the gel formation temperature increased gradually with the increase in DOX-loaded GSH-GG concentration (>1.5%). As an anionic polysaccharide, GG is gelated in the presence of cations, and GSH-GG also preserves this property. Figure 2B–D show that the aqueous solution of DOX-loaded GSH-GG, stored in a lower concentration of Ca^2+^ ions (0.01% *w*/*v*) to form a clarifying solution, acquired viscosity with the increase in Ca^2+^ ion concentration. It was also noted that GSH-GG formed a gel in the presence of Ca^2+^ ions with a concentration of more than 0.025% (*w*/*w*). Similarly, Mg^2+^ exerted an effect on DOX-loaded GSH-GG similar to that of Ca^2+^. However, the sensitivity of DOX-loaded GSH-GG to Na^+^ was relatively poor, which may be attributed to the higher sensitivity of GG to divalent cations [28,29].

### 2.3. Injectability Study

**Figure 3:**
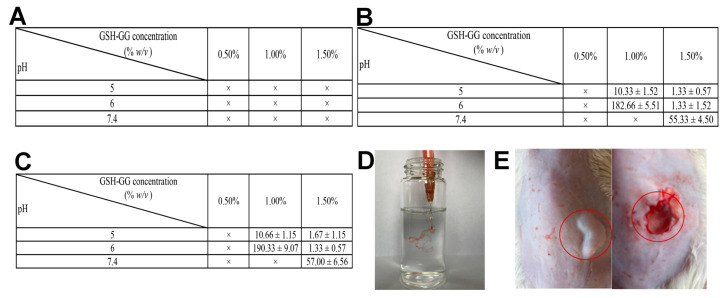
Injectability study of GSH-GG. The time (s) required for DOX-loaded GSH-GG to form a gel after adding (**A**) PBS, (**B**) CaCl_2_ and (**C**) normal saline. (**D**) The gel formation image of DOX-loaded GSH-GG in normal saline. (**E**) The DOX-loaded GSH-GG was injected subcutaneously into the mice (left), and the in-situ gel (right) was observed after the skin was cut open.

The injectable gel can be formed subcutaneously or locally in situ after injection, which is very helpful in avoiding unnecessary surgery on patients receiving tumor treatment [30]. Therefore, the injectability of DOX-loaded GSH-GG gel was studied in vitro and in vivo. The gelation time of DOX-loaded GSH-GG was evaluated in PBS, CaCl_2_, and normal saline (Figure 3A–C). The results displayed a poor gelling property in PBS, possibly pertaining to the low cation concentration in PBS. Conversely, the gel formation occurred faster in normal saline at low pH. For example, the gelation time of 1.5% DOX-loaded GSH-GG increased from 1.67 s at pH 5 to 57.00 s at pH 7.4. DOX-loaded GSH-GG showed the same trend in CaCl_2_, but the gelation rate was faster. When pH was 5, the gelation took about 1.33 s; when the pH rose to 7.4, the gelation time was about 55.33 s. Figure 3D illustrates filamentous gel formation following the injection of DOX-loaded GSH-GG gel into normal saline through a syringe. Next, DOX-loaded GSH-GG was injected subcutaneously into the back of mice, and the skin was cut and observed. The in situ gel formation was apparent at the site of administration (Figure 3E).

### 2.4. In Vitro Release

**Figure 4:**
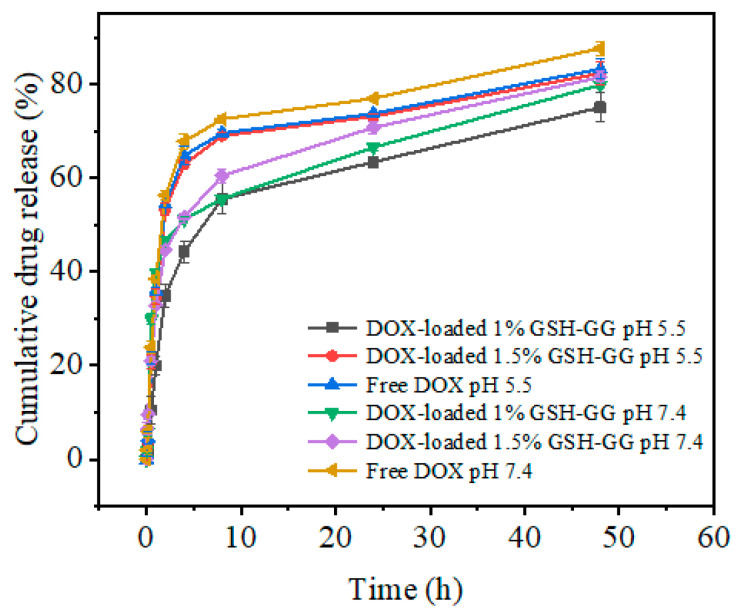
The drug release of free DOX and DOX-loaded GSH-GG under different conditions.

The in vitro drug release curve of GSH-GG loaded with DOX was studied under different pH conditions (physiological pH 7.4 and intracellular lysosomal pH 5.0) at 37 °C (Figure 4). Within 48 h of incubation, DOX-loaded GSH-GG demonstrated a certain sustained drug release effect compared to free DOX and a slower drug release rate under acidic conditions. In fact, very rapid drug release leads to the fast depletion of the drug in the body, intensifying the need for frequent dosing frequency of short time intervals and eventually increasing the treatments’ side effects. In contrast, very slow drug release reduces the drug effect in the target area and provides a great opportunity for the growth and proliferation of damaged cells. Thus, drug release kinetic studies are performed to determine the optimum drug release time at the tumor site for beneficial and effective treatment. In this work, a temperature-ion-pH triple responsive GG was synthesized and utilized in developing DOX-loaded GSH-GG hydrogels. To predict the release mechanism, the obtained in vitro release data of the hydrogel were reviewed via the following four mathematical models: zero-order describes the system where the drug release rate is independent of its concentration, and the process of drug release is constant from the carrier, first-order realizes that release rate is directly proportional to the drug concentration undergoing reaction (the greater the concentration, the faster the reaction), Higuchi, in which the release according to this model occurs in the homogeneous/solid matrix carrier through drug solubility, given that the initial drug concentration is greater than its dissolution rate, and the Korsmeyer–Peppas model that depicts drug release via the diffusion mechanism from polymeric carriers [31]. Various parameters, such as regression coefficients (R^2^) and rate constant factors (k), of all four models were obtained following the analysis of the in vitro drug release data at pH = 7.4 and pH = 5.5. The drug release data were best fitted to the Korsmeyer–Peppas model (Table 1), indicating that DOX release from the GSH-GG carrier was controlled by the diffusion mechanism. This property can take advantage of the tumors’ acidic environment to enable the slow release of the loaded drug, so it can reduce the pain caused by frequent administration [32].

### 2.5. Cytotoxicity Assay

**Figure 5:**
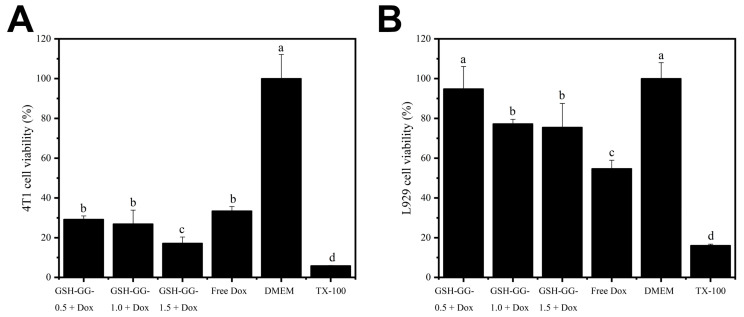
The cytotoxicity of DOX-loaded GSH-GG against 4T1 (**A**) and L929 (**B**) cells. a–d Means in the same row with different letters differ significantly, *p* < 0.05.

The in vitro anticancer effect of DOX-loaded GSH-GG was also tested, and the results are summarized in Figure 5. It is shown in Figure 5A that in the 4T1 cancer cell line, a higher gel concentration (1.5%) of DOX-loaded GSH-GG exerted the highest cytotoxicity effect with an inhibition rate over 80%, whereas 0.5% and 1% gel concentrations demonstrated analogous cytotoxicity. This was an exciting result, which suggested that DOX-loaded GSH-GG at the optimal gel concentration might be a promising DDS for cancer therapy. However, side effects of DDS are another critical issue to be considered in clinical practices [33]. In this regard, we employed normal epithelial cells (L929) to test the cytotoxicity effects of different formulations on normal cells. As shown in Figure 5B, in contrast to cancer cells, all gel formulations showed sharply reduced cytotoxicity on L929 cells. Although reduced cell cytotoxicity was also observed in free DOX, it could be ascribed to the lower drug cellular uptake in normal cells [34]. The cell viability in the gel formulation groups was all significantly higher than that in the free DOX group. These results clearly suggested differential cytotoxicity profiles of DOX-loaded GSH-GG on cancer cells and normal cells. This phenomenon is beneficial for the safe application of DOX-loaded GSH-GG as a promising DDS in vivo and its underlying mechanisms deserve further explorations.

### 2.6. In Vivo Anticancer Assay

**Figure 6:**
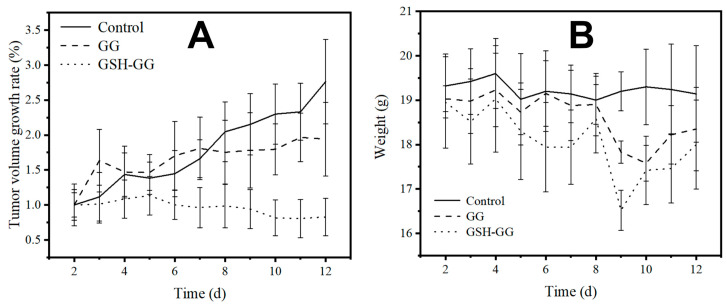
The tumor growth rate (**A**) and body weight (**B**) changes in 4T1 tumor bearing mice treated with DOX-loaded GG and GSH-GG.

The in vivo anti-tumor experiment was performed using 4T1 tumor bearing mice as a model. As shown in Figure 6, due to the controlled release benefits of in situ gels, both DOX-loaded GG and GSH-GG showed enhanced anticancer performance compared to the control group. In particular, the DOX-loaded GSH-GG exhibited the strongest potency, by showing almost complete tumor inhibition at the end of the test. These results suggested that DOX-loaded GSH-GG can provide long-acting benefits and total inhibition of tumor progression, which might be applied as a promising tool for potential clinical applications. The body weight variations in mice during the whole test demonstrated no difference among the three groups, implying the high biocompatibility of GSH-GG for in vivo applications.

## 3. Conclusions

In this study, GG was modified with GSH and employed successfully to construct a new temperature-ion-pH triple responsive GSH-GG gel with reduced gelling temperature, preserved ion-responsive nature, and pH-responsive sustained drug release suited for clinical cancer therapy. Our results demonstrated that DOX could be readily loaded into the matrix of GSH-GG, and the obtained DOX-loaded GSH-GG could exhibit good viscosity, strength, and injectability at an optimized gel concentration of 1.5%, in both in vitro and in vivo conditions. Moreover, DOX-loaded GSH-GG presented sustained drug release compared to free DOX and an especially lower drug release rate under acidic conditions. This was beneficial for its long-acting performance under acidic tumor microenvironments for cancer therapy. In the in vitro cytotoxicity assays, DOX-loaded GSH-GG showed different cytotoxicity on cancer cells (4T1) and normal epithelial cells (L929), which is beneficial for the selective killing of neoplastic cells. Most importantly, a single injection of DOX-loaded GSH-GG caused complete inhibition of tumor progression, due to its long-acting benefits. It also showed high biocompatibility with no significant impact on the subjects’ body weight. In summary, GSH-GG can be applied as a responsive gelling material for delivering DOX as promising cancer therapy.

## 4. Materials and Methods

### 4.1. Materials

GG (GelzanTM CM) was purchased from Sigma-Aldrich (Saint Louis, MO, USA). 1-ethyl-3-[3-(dimethylammino) propyl]carbodiimide (EDAC) was purchased from Adamas-beta (Shanghai, China). 5, 5-dithio-bis(2-nitrobenzoic acid) (DTNB) was purchased from Aladdin (Shanghai, China). GSH was purchased from Acros Organics (Waltham, MA, USA). Dulbecco’s Modified Eagle’s Medium (DMEM), fetal bovine serum (FBS) and DOX were purchased from Shanghai Titan Technology Co., Ltd. (Shanghai, China).

### 4.2. Synthesis of the GSH-GG

Typically, 0.90 g EDAC was added to 30 mL of 1% (*w*/*v*) deacylated GG solution and dissolved by stirring at 25 °C. Then, 0.90 g GSH was added and the pH of solution was adjusted to 5.0 with NaOH (1 M). The reaction mixture was incubated for 3 h. The resulting mixture was acidified with hydrochloric acid (pH = 3) and dialyzed with nitrogen-saturated DI water (intercepting molecular weight: 12–14 kDa) at 4 °C, until the absorbance at 412 nm of the dialysate was not detected using DTNB. The GSH-GG was obtained as a white fibrous solid by lyophilizing.

### 4.3. Gel Formation Assay

The GSH-GG solid fibers were dissolved in DI water at 80 °C, and mixed solutions of different concentrations (0.5%, 1%, 1.5%, *w*/*v*) were prepared. After cooling, 0.2–2 mL normal saline was slowly added to the solutions, and gelation time was recorded. With regard to the gelling standard, after inverting the mixture’s container, the content did not slip off for 15 s [35].

### 4.4. The Preparation of DOX-Loaded GSH-GG

DOX was dissolved in DI water at 37 °C in advance and then mixed into a cooling solution of dissolved GSH-GG solid fibers. The DOX-loaded GSH-GG (gel) was obtained after standing.

### 4.5. Viscosity

The viscosity was determined according to the previous report with some modifications [36]. Different concentrations of DOX-loaded GSH-GG (0.5%, 1%, 1.5%, *w*/*v*) were prepared and preheated to 37 °C. Subsequently, 2–16 mL of normal saline was added dropwise to parallel samples of each concentration. The viscosity was then measured by a rotational viscometer (NDJ-1B, Shanghai Jichang Geological Instrument Co., Ltd. Shanghai, China) using a No. 1–4 rotator at 12–60 r/min.

### 4.6. Rheology of GSH-GG Hydrogel Measurements

The rheological properties of GSH-GG hydrogels with concentrations of 0.5%, 1%, and 1.5% were characterized using a Marvin (Kinexus Pro, Britain) rotational rheometer, equipped with a set of parallel plates with diameters of 20 mm. The scanning frequency range was set to 0.1–100 Hz.

### 4.7. Injectability Studies

The injectability of the gel was determined according to the previous report with some modifications [37]. The injectivity of the DOX-loaded GSH-GG gel was studied both in vitro and in vivo. First, 0.5%, 1%, and 1.5% DOX-loaded GSH-GG (0.5 mL) were injected into PBS, CaCl_2_, and normal saline (pH4, pH5, and pH7.4, 3 mL) via a 1 mL syringe. Then, DOX-loaded GSH-GG was injected subcutaneously into male mice to demonstrate in situ gelation.

### 4.8. In Vitro Adhesion

The smooth glass plate was placed at an angle of 45 to the horizontal plane, and 2 mL GSH-GG of each concentration was dropped onto the glass plate, sliding down naturally, and the time was recorded for the glue drop to slide down 1 cm.

### 4.9. Effect of Temperature on Gelling Behavior of GSH-GG

The effect of temperature on the gelling behavior of GSH-GG was determined using a previously reported protocol with some modifications [38]. DOX-loaded GSH-GG aqueous solutions of different concentrations (1.0–3.0%, *w*/*v*) were prepared. The temperature of the above polymer solution was controlled via a water bath, and its gel state was judged by the inversion method.

### 4.10. Effect of Cations on Gelling Behavior of GSH-GG

The effect of cations on the gelling behavior of GSH-GG was determined using a previously reported protocol with some modifications [39]. DOX-loaded GSH-GG aqueous solutions of different concentrations (0.50–1.5%, *w*/*v*) were prepared. Calcium chloride solutions of different concentrations were added to the polymer solutions prepared above, and the gelation state was judged by the inversion method.

### 4.11. In Vitro Drug Release of DOX-Loaded GSH-GG Hydrogels

The in vitro drug release assay was performed following a previously published protocol [40]. Transdermal apparatus was used to evaluate the release of DOX in GSH-GG. Briefly, free DOX solution (100 μg/mL), 1% DOX-loaded GSH-GG and 1.5% DOX-loaded GSH-GG were put into the transdermal apparatus, and the dialysate was PBS of pH = 5.5 and pH = 7.4, respectively. The ambient temperature of the dialysis was maintained at 37 °C, with constant stirring at 100 rpm. After the diffusion, the medium was removed at different times, and the aliquots were replenished with an equivalent volume of PBS. Then, the concentration of DOX was determined by measuring the fluorescence intensity of the sample using an enzyme labeling instrument at 590 nm.

### 4.12. Cytotoxicity

L929 and 4T1 cells were placed in a 96-well plate with a density of 1 × 10^4^ cells/well, and cultured overnight, respectively. Then, the cells were incubated with 120 μL DOX-loaded GSH-GG (0.5%, 1% and 1.5% *w*/*v*) or free DOX solution for 24 h. The cells were stained with MTT solution for 4 h, and the cell viability was assessed by measuring absorbance at 490 nm with a microplate reader (Multiskan FC, Thermo Scientific, Waltham, MA, USA) [41].

### 4.13. In Vivo Anti-Tumor Effect

The study was conducted according to the guidelines of the NIH Animal Research Facility Orientation Course and approved by the Institutional Review Board (or Ethics Committee) of Shandong University. Female BALB/c mice (18~22 g) were used as the animal models. 4T1 cancer cells were used as cell lines to implant tumors, and DOX was selected as the anti-tumor model drug. To study the anti-tumor effect of free drugs and drug-loaded gels, experimental mice were randomly divided into three groups as shown in Table 2. (*n* = 6 in each group). After the tumor grew to about 100 mm^3^, the experimental mice of Group 2 and Group 3 were injected with 100 μL gel or drug-loaded gel in situ (the content of DOX was 1 mg/mL, the grouping was shown in Table 2). In contrast, the experimental mice of Group 1 did not receive any treatment for two weeks. During the administration period, the body weight of mice in each group was weighed on the electronic scale every day, and the change curve of body weight was drawn. The longest size (L) and widest size (W) of the subcutaneous tumors were measured with a Vernier caliper. The tumor volume was calculated using the formula V = L × W^2^/2, and the tumor growth curve was drawn. Two weeks later, the experimental mice were killed by the cervical dislocation method, and the tumor was stripped off and sectioned [42,43].

### 4.14. Statistical Analysis

The results are expressed as mean ± SD. Statistical comparisons were performed by one-way analysis of variance (ANOVA), followed by Duncan’s multiple range test (DMRT). *p* < 0.05 was considered significant.

## Figures and Tables

**Table 1:** Determination of kinetic parameters and mechanism of DOX released by 1.5% (*w*/*v*) GSH-GG (pH = 7.4 and pH = 5.5).

Conditions	Mathematical Models	R^2^	K	*n*	Release Mechanism
pH = 7.4, 1.5% GSH-GG	Zero-order	0.57611	1.29968	-	
	First-order	0.9254	0.50898	-	
	Higuchi	0.80759	10.6925	-	
	Korsmeyer–Peppas	0.98972	970.346	0.01249	Fickian diffusion
pH = 5.5, 1.5% GSH-GG	Zero-order	0.40511	1.30751	-	
	First-order	0.97956	0.59000	-	
	Higuchi	0.65597	11.2961	-	
	Korsmeyer–Peppas	0.94492	128,942	0.00011	Fickian diffusion

**Table 2:** Grouping of animal experimental mice.

Group	Tumor	Type of Administration
1	+	Saline
2	+	DOX-loaded GG
3	+	DOX-loaded GSH-GG

## Data Availability

The data presented in this study are available upon request from the corresponding author.
